# Management Strategies for POSEIDON Groups 3 and 4

**DOI:** 10.3389/fendo.2019.00614

**Published:** 2019-09-11

**Authors:** Thor Haahr, Carlos Dosouto, Carlo Alviggi, Sandro C. Esteves, Peter Humaidan

**Affiliations:** ^1^Department of Clinical Medicine, Aarhus University, Aarhus, Denmark; ^2^The Fertility Clinic Skive, Skive Regional Hospital, Skive, Denmark; ^3^Hospital de la Santa Creu i Sant Pau- Fundació Puigvert, Obstetrics, Gynecology and Reproductive Medicine, Barcelona, Spain; ^4^Faculty of Medicine, Autonomous University of Barcelona, Barcelona, Spain; ^5^Department of Neuroscience, Reproductive Science, and Odontostomatology, University of Naples Federico II, Naples, Italy; ^6^Consiglio Nazionale Delle Ricerche, Istituto per L'Endocrinologia e L'Oncologia Sperimentale, Naples, Italy; ^7^ANDROFERT, Andrology and Human Reproduction Clinic, Campinas, Brazil; ^8^Department of Surgery, University of Campinas, Campinas, Brazil

**Keywords:** poor ovarian response, Bologna criteria, POSEIDON criteria, controlled ovarian stimulation, blastocyst, pregnancy, ART calculator

## Abstract

In the POSEIDON classification, patients belonging to groups 3 and 4 share the same common feature of a poor ovarian reserve which independently of age renders them at high risk of a poor reproductive outcome. Overall, POSEIDON groups 1–4 constitute approximately 47% of patients attending assisted reproductive technology (ART) treatment. With the increasing delay in childbearing, POSEIDON group 4 seems to increase in numbers now in some centers constituting more than 50% of the total POSEIDON population, whereas group 3 patients constitute approximately 10%. Both POSEIDON groups 3 and 4 patients require special attention as regards pre-treatment strategy, ovarian stimulation, adjuvant treatment, and ovulation trigger strategy in order to optimize the probability of having at least one euploid blastocyst for transfer. Although more evidence is needed, recent advances seem to have increased the reproductive outcomes in the poor prognosis patient. The key to success is individualization in all steps of ART treatment. Herein, we review the recent evidence for the management of POSEIDON groups 3 and 4.

## Prevalence of POSEIDON Groups 3 and 4

The POSEIDON (Patient-Oriented Strategies Encompassing IndividualizeD Oocyte Number) population constitutes 47% of patients referred to Assisted Reproductive Technology (ART) treatment (1). POSEIDON group 3 constitutes 10% whereas POSEIDON group 4 constitutes 55% (1). In a group of Bologna criteria poor ovarian response (POR) patients, the prevalence of POSEIDON group 3 and 4 patients was recently reported to be 24% (13/54) and 76% (41/54), respectively (2). As these patients have a high risk of ending up with no high quality embryos for transfer (3), they often undergo repeated numbers of ovarian stimulations with a subsequent increase in both physical, emotional and financial cost. In this review, we add to the prior work considering POSEIDON classification (4–6) by giving recommendations for clinical management and further research in POSEIDON groups 3 and 4.

## Evidence for Managing POSEIDON Groups 3 and 4 Patients

Although studies in POSEIDON groups 3 and 4 patients are emerging (7, 8), there are currently very few prospective studies comparing different treatment strategies. Hence, the present suggestions for clinical management is mainly based on evidence from patients labeled with POR. In this aspect, it is important to distinguish between studies performed before and after the introduction of the Bologna criteria for POR. Prior to the Bologna criteria, studies used multiple definitions of POR, introducing heterogeneity and subsequently a poor clinical value of the reported results, in particular those of meta-analyses (9). In the latest Cochrane review from 2010 in POR management, it was reported that there is no evidence to support one particular intervention (10). However, Cochrane meta-analyses may not be the optimal tool to evaluate treatment strategies while such strategies are still undergoing development and additional fine tuning (11, 12). In this aspect, and while waiting for better evidence, this review may help clinicians plan how to most optimally manage the poor prognosis patient which is an integral part of daily clinical life.

## Measure of Success

**Figure 1:**
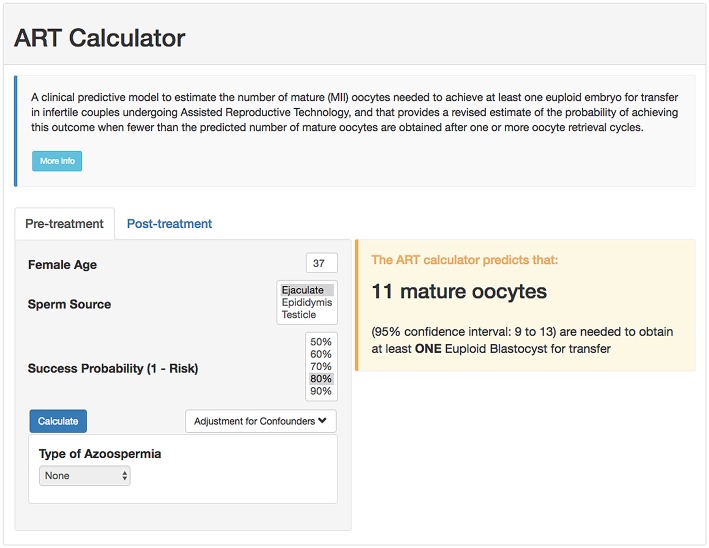
Online calculator to determine the minimum number of mature oocytes required to obtain at least one euploid blastocyst for transfer in infertile patients undergoing IVF/ICSI cycles. The figure shows how the online calculator can be used in an office-based setting. Pre-treatment, clinicians should input the patient age and the sperm source to be used for IVF/ICSI. If the option “Testicle” is marked, then the type of azoospermia should be also defined. The probability of success is set by the user and indicates the chance of having ≥1 euploid blastocyst when the predicted number of mature oocytes is achieved. Its complement is the risk, that is, the chance of having no (zero) euploid blastocysts when the predicted number of oocytes is achieved. Once the button “calculate” is pressed, a text box will pop-up on the right side of the screen, indicating the predicted minimum number of mature oocytes needed for obtaining at least one euploid blastocyst, with its 95% confidence interval (reprinted with permission of the author).

**Figure 2:**
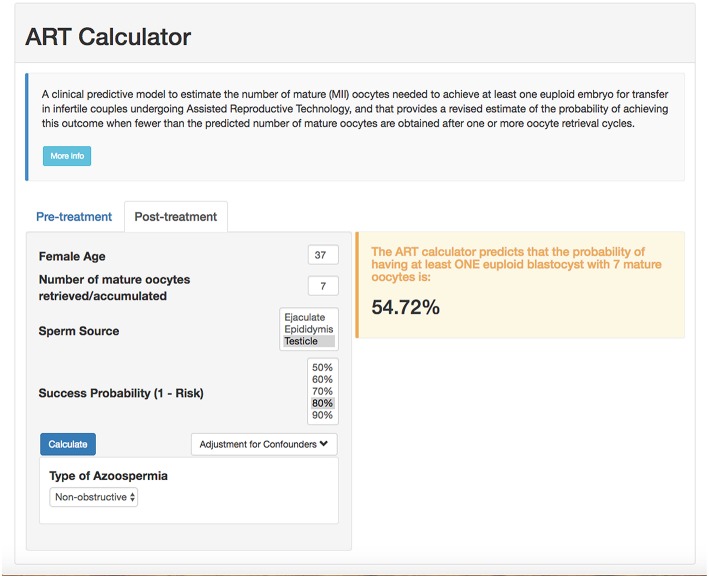
ART online calculator. The figure shows how the online calculator can be used post-treatment, i.e., when fewer than the predicted number of mature oocytes are obtained after one or more oocyte retrieval cycles. Clinicians should input the pre-treatment information and the actual number of mature oocytes collected or accumulated. The probability of success is set by the user; it reflects the chance that the estimation is correct given the number of oocytes input. Once the button “calculate” is pressed, a text box will pop-up on the right side of the screen, indicating the predicted probability of achieving ≥1 euploid blastocyst with the number of mature oocytes available (reprinted with permission of the author).

According to the POSEIDON concept, the measure of success is to increase the probability of having at least one euploid blastocyst for transfer in the individual patient (6). Recently, a predictive tool so-called “ART Calculator” was launched to estimate the number of oocytes needed to have at least one euploid blastocyst for transfer, available on http://www.members.groupposeidon.com/Calculator/. This calculator provides the estimation mentioned above based on a number of predictors such as female age and type of sperm, which were found to be relevant concerning blastocyst euploidy, see Figure 1 (1, 13). Thus, using mathematical equations and the age-related probabilities of a blastocyst being euploid per mature oocyte as a function of sperm source, the ART calculator makes two types of predictions automatically, one using pre-treatment information to estimate the minimum number of mature oocytes to achieve at least one euploid blastocyst, and another based on the actual number of mature oocytes collected/accumulated to estimate the chances of having a euploid blastocyst using that oocyte cohort for IVF/ICSI, see Figure 2. Apart from guiding the clinician in individualized management, the ART calculator constitutes an ideal tool to counsel patients about their prognosis when embarking on ART treatment, and subsequently, at the time of oocyte retrieval where some patients might be counseled to go directly to a luteal phase stimulation in order to increase the chances of having at least one euploid blastocyst for transfer (13). As an example, the ART calculator estimates that at least 6–9 and 10–15 mature oocytes are needed to obtain one euploid blastocyst for transfer in POSEIDON groups 3 and 4 patients aged 33 and 36 years-old, respectively, assuming a 90% probability of success in the estimations when ejaculated sperm is used for IVF/ICSI. Hence, planning of the most optimal ovarian stimulation regimen is of paramount importance to achieve the highest success rate.

## Ovarian Stimulation in POSEIDON Groups 3 and 4

### Natural Cycle or Stimulation With Exogenous Gonadotropins

Previously, some authors expressed concern that stimulation *per se* would increase embryonic aneuploidy rates, suggesting that natural cycle IVF might be an option for the POR patient (14, 15). However, abundant evidence does not support this concern neither in young oocyte donors nor in PGS IVF-ET patients (16–19). Moreover, natural cycle IVF results in extremely low live birth rates in the POR patient with a reported live birth rate per cycle of only 2.6% and a cumulative live birth rate of only 7% after six natural IVF cycles in Bologna POR patients (20). Similarly, extremely low live birth rates after natural cycle IVF have been corroborated by others (21). In contrast the largest RCT aligned with the ESHRE Bologna POR criteria reported a live birth rate per cycle of 11% using a combination of a long gonadotropin releasing hormone agonist (GnRHa) down-regulation protocol and daily gonadotropin dosing with 300 IU recombinant FSH and 150 IU recombinant LH (22). Recently, combining follicular and luteal phase stimulation in the same ovarian cycle two trials reported ongoing pregnancy rates above 20% per DuoStim cycle in poor prognosis patients (2, 23). Thus, ovarian stimulation rather than natural cycle should be the preferred first line treatment in the poor prognosis patient with a poor ovarian reserve.

### Stimulation Protocol

A meta-analysis in non-Bologna criteria POR patients explored the optimal GnRH analog treatment (24). From this analysis, it was concluded that there was no significant difference in clinical pregnancy rates comparing the long GnRHa down-regulation protocol to the GnRH antagonist protocol, although the trend favored the long GnRHa down-regulation protocol. Later, a study published in Bologna POR patients reported that the long GnRH agonist protocol, albeit non-significantly, increased the number of mature oocytes by one oocyte as compared to the GnRH antagonist protocol (25). Moreover, the cancellation rate was significantly lower for the long GnRHa protocol. The biological plausibility for this finding may be follicular synchronization obtained after downregulation, which is paramount for the poor ovarian reserve patient as this patient usually has an increased late luteal FSH level, promoting early recruitment of the leading follicle, which in turn will suppress the early growth of the few other follicles residing in the ovary. This inhibitory effect on endogenous FSH—and early recruitment—can also be achieved in GnRH antagonist cycles, using short term daily estradiol 4 mg, or oral contraceptives for 12–16 days as pre-treatment without compromising reproductive outcome as compared to the long GnRHa down-regulation protocol (26, 27). As one more oocyte increases the live birth rate (LBR) by approximately 5% (28, 29), a long GnRH agonist down-regulation protocol or a “primed” GnRH antagonist protocol as mentioned above should be considered first line treatment for the poor prognosis patient. A recent retrospective study in POSEIDON groups 3 and 4 patients reported that a higher live birth rate per initiated cycle can be achieved in group 3 patients by using hMG in GnRHa down-regulation protocol as compared to hMG in GnRH antagonist protocol (7/54 = 13.0% vs. 78/283 = 27.6%, *p* = 0.024) (7). This effect was not noted in POSEIDON group 4 patients. However, a retrospective analysis of 999 poor prognosis patients (defined as AFC < 11 and AMH < 1.1 ng/ml) in the long down-regulation protocol and comparing a rLH + rFSH regimen to hMG showed that rLH + rFSH was superior to hMG regarding the clinical pregnancy rate per started cycle (12.5 vs. 8.1%, *P* < 0.02) (30). Interestingly, this effect was even more pronounced in the patients with AFC <4 (10.2 vs. 1.5%, *P* < 0.01). Another protocol is the so-called mild stimulation protocol (31, 32), but this approach is poorly defined most often involving the GnRH antagonist protocol using low dose gonadotropin stimulation compared to a long GnRH agonist protocol with higher doses of gonadotropin (33). Although recommended in the American clinical guideline for POR (34), mild ovarian stimulation for POSEIDON groups 3 and 4 is an approach not in line with the POSEIDON stratification as discussed extensively in the paragraphs on natural cycle and FSH dosing.

The recent advances in dual stimulation (“DuoStim”) represents an interesting solution to accumulate embryos (blastocysts) within a short time span in order to obtain the number of blastocysts needed to increase the probability of having at least one euploid blastocyst for subsequent elective frozen embryo transfer (eFET) (35–38). In a recent publication, Vaiarelli et al. (23) reported that poor prognosis patients (essentially POSEIDON group 4) undergoing a single “DuoStim” cycle resulted in a total of 65.5% (203/310) of patients having at least one euploid blastocyst for transfer (23). Following single euploid blastocyst transfer the ongoing pregnancy rate per transfer was similar comparing blastocysts obtained from follicular phase stimulation and blastocysts obtained from luteal phase stimulation, 39.5% (32/81) and 49.4% (41/83), respectively (23). Although the study excluded patients with no response to DuoStim (43/353), an ongoing pregnancy rate per DuoStim cycle of 20.7% (73/353) in POSEIDON group 4 patients can be considered highly successful in this difficult patient group. Another recent study used the combined advantages of Corifollitropin alfa and Duostim in Bologna criteria patients (*N* = 54), 24% (13/54) and 76% (41/54) were POSEIDON group 3 and 4 patients, respectively. In this study, authors reported an ongoing pregnancy rate per DuoStim cycle of 20.4% (11/54) (2). Hence, evidence suggest that even in poor prognosis patients ongoing pregnancy rates of around 20% can be achieved. However, there are currently no results from prospective randomized trials comparing DuoStim to two conventional stimulation cycles with cumulative live birth rate and time to live birth as end points. Importantly, in DuoStim a freeze-all policy is mandatory, which includes additional manipulations with biological material and costs for the patient or the health care system. Until further, we have to await the results of registered ongoing trials before final conclusions can be made.

### Choice of Gonadotropin

A Cochrane meta-analysis covering the normogonadotropic IVF/ICSI population concluded that the type of gonadotropin should be based on availability, convenience and costs (39). Likewise, a large survey involving 314 centers from 73 countries worldwide concluded that the majority of respondents (62.2%) did not believe that there was a difference in efficacy between urinary (u) FSH and rFSH preparations and that the choice of gonadotropin was most often based on the individual preference of the clinician (40). Despite no significant results comparing all types of uFSH with rFSH in normogonadotrophic women (28 trials, 7,339 couples, odds ratio (OR) 0.97, 95% CI 0.87–1.08) (39), a sub analysis observed that hMG was superior to rFSH as regards live birth rate per woman (11 trials, *N* = 3,197, OR 0.84, 95% CI 0.72–0.99). However, a recent meta-analysis of 70 prospective studies considering all gonadotropin combinations and all ART outcomes, reported that recombinant FSH alone resulted in greater number of oocytes than hMG or rFSH+rLH (41). The addition of LH activity was useful to reduce the amount of FSH needed and to improve pregnancy outcome, but only if LH activity was provided by rLH rather than hCG. In the context of this review, the question is whether these results can be extrapolated to poor prognosis patients and, admittedly, the results are difficult to interpret. When the effectiveness of the gonadotropin regimen is the focus of the investigation, the primary endpoint should also include the ovarian response, which is a critical measurable parameter of gonadotropin action (42). By contrast, pregnancy is the final result which is influenced by a multitude of factors, including endometrium receptivity, sperm factors, etc. In this regard, high quality evidence overwhelmingly indicates that recombinant FSH is superior to urinary FSH and hMG as a means to increase the oocyte yield (43–47). Since the POSEIDON criteria relies on the individualized oocyte number to increase the likelihood of having at least one euploid blastocyst for transfer, it seems sound to conclude that recombinant FSH, used alone or combined with recombinant LH, is the natural choice in Poseidon group 4 patients. The use of gonadotropin regimens combining recFSH and LH activity supplementation by recLH in Poseidon group 4 might offer additional clinical benefit, as discussed in the next section, owing to a fine-tuned modulation of the PKA pathway and proliferative/antiapoptotic signals, unlike hCG (42). In conclusion, hMG does not seem to add any clinically significant benefit as regards reproductive outcomes in the GnRH antagonist protocol, and likewise added LH activity in the long GnRHa down-regulation protocol seems to be better covered by r-LH than by hMG.

Another agent for ovarian stimulation is Corifollitropin alfa which has the pharmacokinetic advantage of a rapid increase in the FSH serum level which optimizes early recruitment and increases the number of pre-ovulatory follicles (48). In a RCT including Bologna PORs only, there was no significant difference in live birth rate after fresh embryo transfer, however, significantly more embryos were cryopreserved in the group treated with Corifollitropin alfa followed by hMG as compared to a rFSH only regimen which hypothetically would increase the cumulative live birth rate (49). From a POSEIDON point of view, it is important to achieve more embryos in order to maximize the chance of having one euploid blastocyst for transfer, however, a larger sample size would be needed to reach statistical significance as regards live birth rates (49).

### FSH Dosing: Individualization or One Size Fits All?

Recently, the OPTIMIST trial reported that a starting dose of 150 IU FSH (91% used rFSH) provided a similar cumulative LBR after 18 months follow-up as compared to individualized dosing with either 225 or 450 IU FSH in poor prognosis patients (*N* = 511), who were defined as having an antral follicle count of either 8–10 or <8, respectively (50, 51). Subsequently, the study was heavily criticized by many clinical researchers and for a multitude of reasons (52–54). First of all, the definition of poor prognosis was not in line with neither the ESHRE Bologna nor the POSEIDON criteria (52). Secondly, the individualized dosing significantly reduced cycle cancellation and increased the number of good quality embryos for transfer and, finally, the 18-month follow-up period for cumulative live birth rate was criticized for not sufficiently covering supernumerary FET cycles (54). In fact, to show an increase from 20 to 25% in LBR, more than 2,000 patients should have been randomized in order to achieve significant results (53). Hence, the conclusion of the OPTIMIST trial suffered from many shortcomings and, in our opinion, the current best practice in managing poor prognosis patients should be to individualize the ovarian stimulation in order to increase the oocyte yield which is the only key to optimize LBR as seen in large cohort studies (29, 55, 56). In fact, a pivotal study by Sunkara et al. (*N* = 400,135 cycles) found that increasing the oocyte yield from 2 to 3 resulted in a 25% relative increase in LBR across all age groups (29). Thus, results from large databases with LBR as outcome have a significantly higher clinical value as compared to small and underpowered studies which came to the conclusion that a higher oocyte number does not lead to a higher number of good quality embryos (57) As regards the maximum daily FSH dose, it was shown that rFSH dosing above 300 IU rFSH daily does not seem to increase the LBR (58). In fact, a large retrospective study (*N* = 658,519) reported that daily dosing above 300 IU of FSH (including both uFSH and rFSH) significantly decreased the odds of a live birth (59).

## Adjuvants to Ovarian Stimulation

Over the years many adjuvants to standard ovarian stimulation have been proposed to increase LBR for the POR patient. In this paragraph we focus on relevant adjuvant therapy where evidence is relatively extensive; thus, excluding e.g., use of platelet enriched plasma, mitochondrial transfer and stem cells treatment where evidence is based primarily on case series.

### Androgens

Pretreatment with androgens has been used for the POR patient in several trials. This approach could be considered for Poseidon groups 3 and 4 where, independently of age, the ovarian reserve is reduced and POR is expected. The main biological evidence from the primate model is that androgens induce FSH receptors on granulosa cells (60), which in turn increases the recruitability and growth of pre-antral and antral follicles, through the IGF-1 system (61, 62). In 2012, two independent meta-analyses reported a significant positive effect of transdermal testosterone on the LBR of POR patients (63, 64). However, only a total of 82 patients and 113 patients were included in the intervention arm of the respective meta-analyses, which again included studies performed prior to the Bologna criteria. In another meta-analysis of four RCT's and 2 observational studies including a total of 528 patients, Zhang et al. (65) reported that long-term DHEA treatment, the precursor of testosterone, had a significant positive effect on the LBR of POR patients as compared to controls (RR 1.87, 95% CI, 1.22–2.88) (65). Similarly, the latest Cochrane meta-analysis reported moderate quality evidence supporting that DHEA and testosterone pre-treatment may improve LBR in POR patients (66). Although basic scientific and recent clinical evidence seems to support the use of androgen pre-treatment in POR, a recent commentary argued that the “androgen chapter” needs further study before recommendations can be made (67). Especially, the dosage and the timing of pre-treatment needs to be further elucidated; hence an international clinical research group designed the so-called TTRANSPORT TRIAL for Bologna POR patients (Clinicaltrial.gov identifier NCT02418572), evaluating androgen pre-treatment exceeding 60 days, and using a daily dose of 5.5 mg transdermal testosterone. This study designed to include a large population of Bologna POR patients uses androgen pre-treatment in a daily physiological dose and for an extended time compared to previous trials, taking the time needed for folliculogenesis into account. The results of this trial -when completed- could help clarify the clinical utility of pre-treatment with androgens in poor prognosis patients.

### LH Supplementation

The physiological rationale for LH supplementation is primarily based on the “two cell two gonadotropin” concept (68, 69), in which LH supplementation stimulates the conversion of cholesterol into androgens in the theca cell, thus, increasing endogenous intra-ovarian androgen production and follicular growth. On one hand, androgens (i) stimulate FSH receptor expression on granulosa cells (60) (ii) act synergistically with IGF1 for the growth of the follicle (62) and in animal models increase the number of pre-antral and antral follicles (70). On the other hand, LH binding to granulosa cell LH receptors–expressed from the mid-follicular phase onwards- sustains FSH dependent granulosa cell activities, including aromatase induction, release of growth factors and regulates final follicle/oocyte maturation (71, 72). To study the possible clinical effect of rLH supplementation Lehert et al. (73) published a meta-analysis based on 6,443 cycles in normal and poor prognosis patients (non-Bologna criteria) who were supplemented or not with rLH (73). Importantly, in that analysis it was not possible to distinguish between hypo responder and POR patients. While rLH supplementation improved clinical pregnancy rates by 9% (NS) in the overall population, the effect was more pronounced in PORs with a relative risk (RR) of 1.30 (95% CI, 1.01–1.67). Recently, Humaidan et al. (22) published the results of the largest RCT in poor prognosis patients aligned with the ESHRE Bologna criteria and POSEIDON group 4 criteria. In this trial, a total of 939 patients were randomized to either a fixed daily dose of either 300 IU rFSH plus 150 IU r-LH or rFSH 300 IU alone (22). The results indicated no significant differences between groups regarding LBR. However, a *post-hoc* analysis, stratifying patients into mild, moderate or severe POR observed that the moderate and severe PORs benefitted significantly from 150 IU rLH supplementation in terms of a higher LBR and a lower total pregnancy loss (22). Finally, two more recent systematic reviews indicated that rLH supplementation is beneficial in women with hypo-response and in women 36–39 years of age, reinforcing the idea of testing this approach in Poseidon group 4 (74, 75).

### Growth Hormone

Growth Hormone (GH) has been explored therapeutically in ART for more than 30 years. The biological rationale for its use is that GH itself has a synergistic effect to that of FSH on follicular development and also through its downstream mediator, Insulin-like Growth Factor 1 (IGF-1), as seen in animal models (76, 77). All models which block or impair the action of GH, result in a delay in puberty, a significant reduction on litter size and a delay in the exhaustion of the follicular pool (78). Subsequent microscopic examination of the ovaries in these animal models shows an increase in primordial and primary follicles and a decrease in the number of growing antral and pre-ovulatory follicles (78–80). Knock-out female mice failed to ovulate either spontaneously or under the influence of gonadotropins, proving the importance of GH and IGF1 in increasing the sensitivity to gonadotropins during the whole process of selection and follicular growth to ovulation (81). Until now, scientific evidence suggests that adjuvant treatment with GH for POR patients in IVF leads to a higher number of oocytes retrieved and a lower gonadotropin consumption (82–84). However, meta-analyses until now failed to show differences in LBR, perhaps as a result of most trials being underpowered and using different definitions for POR. Moreover, there is high interstudy variability regarding the route, timing and dose of GH administration. The general pattern has been to explore GH adjuvant treatment using the same rationale as for androgen supplementation i.e., pre-treatment for some weeks before stimulation to hypothetically increase the number of recruitable follicles. In this line, a recent double-blind, placebo-controlled randomized trial was performed in 10 centers throughout Australia and New Zealand in POR patients, however, and importantly not aligned with the Bologna or the POSEIDON criteria (85). After 4 years that study was stopped after randomization of a total of 130 patients. Unlike other studies, no statistical differences were reported between groups regarding the mean number of oocytes retrieved (5 vs. 4, rate ratio 1.25, 95% CI 0.95–1.66) and the chance of reaching embryo transfer [53/61 [86.9%] vs. 42/51 [82.4%], OR 1.42, 95% CI 0.50–4.00]. However, results from this study should be interpreted with caution as the study was pre maturely stopped and as such was underpowered.

### Coenzyme Q10 (CoQ10)

CoQ10 pre-treatment for 60 days prior to ovarian stimulation was very recently investigated in a RCT in POSEIDON group 3 patients (*N* = 169 patients) (8). The hypothesis was that CoQ10 would reduce mitochondrial oxidative stress and thus, improve oocyte competence. The study showed a significant difference in the CoQ10 supplemented group regarding number of oocytes retrieved [4 (mean), IQR 2–5] as compared to controls [2 (mean), IQR 1–2], *p* = 0.002, despite the fact that significantly less FSH was consumed in the CoQ10 supplemented group. In addition, the CoQ10 group had more high-quality day 3 embryos defined as embryos that reached 6 to 8-cell stage with cytoplasmic fragmentation occupying <10% of the embryo surface and had equal size blastomeres. The major limitation, however, was the lack of a placebo group. More studies are definitely needed in the area of pre-treatment with CoQ10, including antioxidants in general and specifically for POSEIDON group 3 and 4 patients. Importantly, CoQ10 and other antioxidants are promising adjuvants keeping in mind that they seem to cause no or very limited adverse reactions and side effects (8).

## Ovulation Trigger Strategy

In a recent review (86), the subject of individualized ovulation triggering (OT) was covered in detail. For the present review we extract the important message that achieving the maximum number of mature oocytes can be improved not only by the use of an individualized COS protocol, but also by individualizing the OT strategy. The key for success when using an OT agent is to reach an optimal LH activity level after trigger, resulting in the retrieval of more than 75% mature oocytes and without increasing the risk of OHSS development (87). A previous cycle with a low follicle:oocyte ratio (FOI) could reflect lack of an appropriate follicular response to trigger which could be associated to ovarian aging, poor ovarian reserve or even to mutations of the LH receptor (5, 86, 88). However, a low FOI can be largely improved by carefully considering the OT strategy.

### Human Chorionic Gonadotropin

Human chorionic gonadotropin (hCG) has been used as a surrogate to LH for more than 30 years. Both gonadotropins stimulate the LH receptor due to molecular similarities (89); nevertheless hCG is characterized by having a longer half-life compared to LH (90) and this fact conditions the physiology of the corpora lutea and luteal phase hormonal profile. Using hCG as OT agent ensures an action at the level of the follicle regardless of the pituitary status and hCG trigger with a standard luteal phase support has been shown to yield comparable reproductive outcomes as compared to GnRHa trigger and a modified luteal phase support policy (11).

### GnRHa

GnRHa is a synthetic peptide that interacts with the GnRH receptor releasing LH and to a lesser extent, FSH after activation. In a GnRH antagonist cotreated-cycle, a bolus of GnRHa displaces the GnRH antagonist from the receptor which induces a flare of LH and FSH and subsequently, oocyte maturation and ovulation (91). The amount of LH (and FSH) secreted after GnRHa trigger is significantly reduced in comparison with the natural cycle (92) which leads to implantation failure and early pregnancy loss after fresh embryo transfer, when using a standard LPS, only (93). However, good quality oocytes and embryos were obtained after GnRHa as well as after hCG triggering (94). Moreover, significantly more MII oocytes and embryos were obtained after GnRHa trigger as compared to hCG trigger in a recent retrospective analysis in cancer patients undergoing COS and cryopreservation (95). This finding was supported by a recent systematic review and meta-analysis, in which two RCTs showed a significant increase in the number of good quality embryos after GnRHa trigger as compared to hCG trigger, MD 0.94, 95% CI 0.01, 1.87 (11).

### Combination of OT Agents

OT strategies such as “dual trigger” and “double trigger” have been explored mainly in patients with a low FOI in a previous cycle or a low proportion of mature oocytes and these strategies have been suggested to improve IVF outcomes, to some extent overcoming impairment in follicular function, oocyte meiotic maturation and cumulus expansion (86). Dual trigger is defined as the combined use of GnRHa and a low-dose of hCG, administered simultaneously (96). In contrast, Double trigger is defined as the administration of GnRHa and hCG for OT at 40 and 34 h, respectively, prior to oocyte retrieval (97). Both strategies combine the advantages of GnRHa and HCG: the direct intrafollicular LH activity mediated by hCG, the simultaneous induction of an endogenous FSH surge mediated by GnRHa, and the support of the early luteal phase LH activity mediated by hCG (98). Double trigger adds the aspect of prolonging the interval between OT and the oocyte retrieval which has been described as a strategy to increase the maturity rate of retrieved oocytes. The physiological rationale being that some patients may need a longer exposure time to the OT agent to allow cumulus expansion and detachment of the oocyte (99). However, the evidence for the use of double trigger in patients with low oocyte/follicle yield, low M-2 rate or poor responders is very limited, reported by 2 groups, only, both from Israel (87, 97, 98, 100); thus, awaiting confirmation by further large scaled RCTs. Importantly, the number of cycles included in these series was 1, 12, 8, and 33, only.

## How to Tailor the Most Optimal ART Treatment Encompassing the Different Tools Mentioned to Achieve at Least one Euploid Blastocyst for Transfer

**Figure 3:**
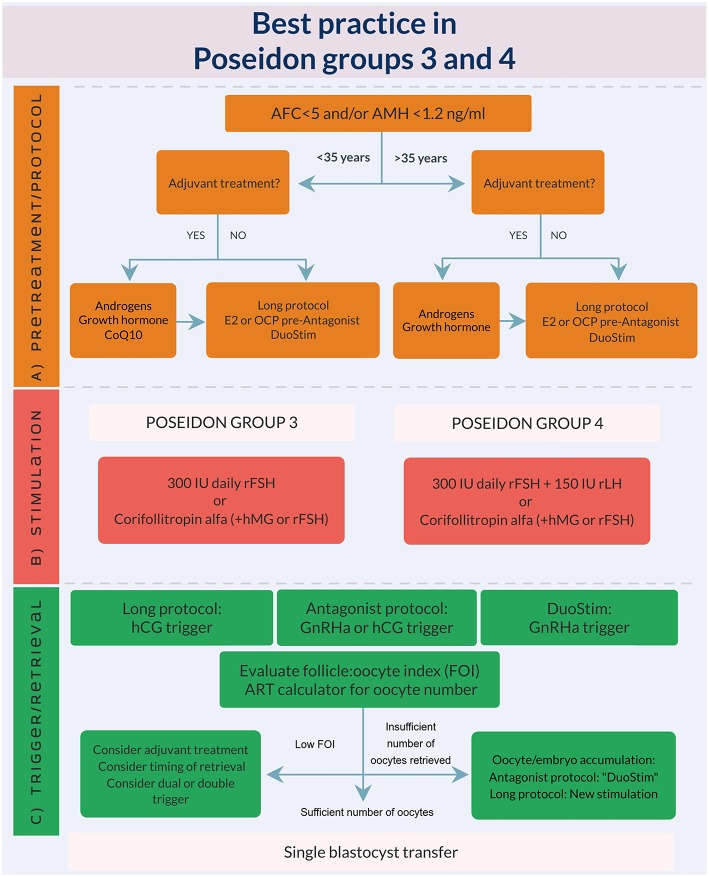
Best practice in POSEIDON groups 3 and 4. **(A)** Pre-treatment is rarely the first option in poor prognosis patients, but in case of unsuccessful ovarian stimulation, i.e., inadequate ovarian response, pre-treatment should be considered. The choice should rely on availability, clinical experience and patient preference. Stimulation protocol might start using GnRH antagonist co-treatment keeping in mind the possibility of converting to DuoStim to achieve the individualized oocyte number (according to the ART calculator). Otherwise a long GnRHa protocol should be considered first choice. **(B)** Ovarian stimulation strategy: First choice in Poseidon group 3 is the GnRH antagonist cycle with either 300 IU daily of rFSH alone or Corifollitropin alfa followed by either rFSH or hMG. In POSEIDON group 4 patients, rLH (75–150 IU daily) should be added from day one of stimulation unless the combination of Corifollitropin alfa and hMG was chosen. The GnRH antagonist cycle allows use of Duostim, unlike the long-agonist GnRH analog. **(C)** Ovulation trigger strategy: In the long GnRHa down-regulation protocol hCG is mandatory as ovulation trigger, whereas GnRHa is mandatory in the follicular phase stimulation of the DuoStim protocol. All trigger agents can be used in the luteal phase stimulation. In non-DuoStim GnRH antagonist cycles, the choice of trigger between GnRHa and hCG should rely on the embryo transfer strategy (fresh or frozen), patient characteristics (e.g., hypo-hypo) and clinical experience. In cases with a low FOI as determined on trigger day, clinicians should consider pre-treatment including short term estrogen therapy or OCP for synchronization of the follicles prior to stimulation, adjuvant LH activity during stimulation, or changing trigger strategy to either dual or double trigger. In case of an insufficient number of oocytes retrieved as determined by the ART calculator, the probability of transferring a euploid embryo should be discussed with the patient to counsel whether an immediate transfer or a new stimulation should be suggested.

Based on the abovementioned evidence, we developed an expert opinion algorithm on how to manage POSEIDON group 3 and 4 patients, see Figure 3. As explained earlier, the suggestions for management is based on “very poor evidence” in terms of GRADE (Grading of Recommendations Assessment, Development and Evaluation). Thus, more research is needed and the suggested recommendations should preferably be used in future RCT‘s or at least clinicians should have retrospective database capture of their results. Despite the poor evidence until now, we believe our suggestions represent current best practice.

## Conclusions

Poor prognosis patients challenge IVF clinicians every day. Herein, we extracted and discussed best practice for these patients. Although more research is needed to make firm clinical recommendations, it is interesting that the treatment concepts discussed herein resulted in ongoing pregnancy rates above 20% per cycle (Duostim) for POSEIDON groups 3 and 4. Future trials investigating pre-treatment strategy, ovarian stimulation strategy and ovulation trigger strategy are warranted and should be based on a more detailed patient stratification such as suggested by the POSEIDON Group.

## Author Contributions

The style and concept were developed by TH and PH. TH produced Figure 3 using visme.co with critical revisions from all authors. All authors contributed to writing the manuscript, contributed with critical review and discussions regarding the final version of this review, and accepted the submission of this manuscript for publication.

### Conflict of Interest Statement

TH received honoraria for lectures from Merck and Ferring. PH received unrestricted research grants from MSD, Merck, and Ferring as well as honoraria for lectures from MSD, Merck, Gedeon-Richter, Theramex, and IBSA. SE received honoraria for lectures from Merck, Lilly, Gedeon-Richter, and Besins. CA received unrestricted research grants from Merck honoraria for lectures from Merck, Ferring and IBSA. PH, SE, and CA are cofounders of the POSEIDON criteria. The remaining author declares that the research was conducted in the absence of any commercial or financial relationships that could be construed as a potential conflict of interest.
